# Psychological distress, wellbeing and resilience: modelling adolescent mental health profiles during the COVID-19 pandemic

**DOI:** 10.1007/s44192-024-00071-8

**Published:** 2024-05-23

**Authors:** Sarah Butter, Mark Shevlin, Jilly Gibson-Miller, Orla McBride, Todd K. Hartman, Richard P. Bentall, Kate Bennett, Jamie Murphy, Liam Mason, Anton P. Martinez, Liat Levita

**Affiliations:** 1 School of Psychology, Ulster University, Cromore Road, Coleraine, BT52 1SA Northern Ireland; 2 School of Education, University of Sheffield, Sheffield, England; 3 Department of Social Statistics, University of Manchester, Manchester, England; 4 Department of Psychology, University of Sheffield, Sheffield, England; 5 Department of Psychology, University of Liverpool, Liverpool, England; 6 Division of Psychology and Language Sciences, University College London, London, England; 7 School of Psychology, University of Sussex, Falmer, England

**Keywords:** COVID-19, Adolescents, Young people, Mental health, Resilience, Latent variable modelling

## Abstract

There has been concern about adolescent mental health during the pandemic. The current study examined adolescent mental health during the initial phase of the COVID-19 pandemic in the UK. Using indicator of psychological distress, wellbeing and resilience, latent profile analysis was used to identify homogeneous mental health groups among young people aged 13–24 (*N* = 1971). Multinomial logistic regression was then used to examine which sociodemographic and psychosocial variables predicted latent class membership. Four classes were found. The largest class (Class 1, 37.2%) was characterised by *moderate symptomology and moderate wellbeing*. Class 2 (34.2%) was characterised by *low symptomology and high wellbeing,* while Class 3 (25.4%) was characterised by *moderate symptomology and high wellbeing.* Finally, Class 4 was the smallest (3.2%) and was characterised by *high symptomology and low wellbeing.* Compared to the *low symptomology, high wellbeing* class, all other classes were associated with less social engagement with friends, poorer family functioning, greater somatic symptoms, and a less positive model of self. A number of unique associations between the classes and predictor variables were identified. Although around two-thirds of adolescents reported moderate-to-high symptomology, most of these individuals also reported concurrent moderate-to-high levels of wellbeing, reflecting resilience. Furthermore, these findings demonstrate how a more comprehensive picture of mental health can be gained through adopting a dual-continua conceptualisation of mental health that incorporates both pathology and well-being. In this way, at-risk youth can be identified and interventions and resources targeted appropriately.

**Supplementary Information:**

The online version contains supplementary material available at 10.1007/s44192-024-00071-8.

## Introduction

Adolescence is a critical developmental period for mental health, with most mental disorders developing prior to age 25 years [1–3]. Against this backdrop of rapid biological, cognitive and social changes, there has been concern that the COVID-19 pandemic could irrevocably damage young people’s mental health [4–7]. These concerns have been supported by research reporting increases in psychopathology among adolescents compared to pre-pandemic levels [8–10], although, these initial elevated levels declined after the peak of infections during the first wave, as the pandemic continued [11–13]. Lifestyle, education and economic disruptions, rather than health risks to self and others, were the strongest drivers of psychological distress for young adults during this time [14].

Yet, scholars have increasingly acknowledged that the absence of psychological distress does not necessarily equate to good mental health [15, 16]. Moreover, mental health models have also shifted to incorporate and emphasise positive wellbeing when conceptualising mental health [17, 18]. While traditional models have considered wellbeing and common mental health disorders as lying at opposite ends of the same spectrum [19], contemporary models posit that they instead reflect distinct continua; they are separate but related constructs, which together encompass ‘mental health’ [18, 20, 21]. Within this ‘dual continua’ or ‘dual factor’ model, levels of both wellbeing and distress can vary and as such, a number of distinct mental health groups have been classified among adolescents, including, ‘complete mental health’ (low psychopathology, high wellbeing), ‘vulnerable’ (low psychopathology, low wellbeing), ‘troubled’ (high psychopathology, low wellbeing) and ‘symptomatic but content’ (high psychopathology, high wellbeing), e.g., [22]. Studies which have utilised empirically-driven methods, such as latent profile analysis (LPA), to classify adolescent mental health groups have reported more nuanced results; including the absence of a ‘vulnerable’ group [23], the presence of a ‘moderately mentally healthy’ group [24] and more than one ‘symptomatic but content’ group emerging [25].

Unfortunately, however, the majority of studies examining adolescent mental health during the COVID-19 pandemic have largely relied on measures of psychopathology (e.g., anxiety, depression, stress) as indicators of mental health, with comparatively fewer studies examining corresponding wellbeing [26]. Furthermore, the dual continua model provides an opportunity to assess adolescents’ response to adversity [27], with few studies specifically examining mental health profiles in this context, e.g., [28]. Thus, rather than solely focussing on psychopathology, which provides a ‘narrow view’, by conceptualising mental health as both a product of wellbeing and symptomology, a more complete and comprehensive picture of adolescent mental health can be achieved [18, 27]. The aim of the current study, therefore, was to model patterns of mental health (including both distress and wellbeing indicators) among young people (aged 13–24 years) during the initial stages of the COVID-19 pandemic. Additionally, a range of sociodemographic and psychosocial variables were used to predict class membership.

## Methods

### Sample

Data for the current study were collected as part of The COVID-19 Psychological Research Consortium (C19PRC) Study. The study was designed to measure, assess, and monitor the adult (aged 18 + years) population’s psychological and social adjustment to the COVID-19 pandemic. However, to better understand the consequences of the pandemic on young people, an ‘add-on’ survey, aimed specifically at young people, was conducted separately. Data collection took place between 21 and 29 April 2020, approximately one month after the first nationwide lockdown was imposed in the UK and during a peak in positive COVID-19 cases and deaths in the UK [29]. During this time, a Stay at Home Order was in place, banning all non-essential contact and travel. Schools closed for in-person teaching (with exception of children of key workers and vulnerable children) and was replaced by online learning. Young people were recruited via convenience sampling by the survey company Qualtrics. Qualtrics partners with over 20 online sample providers to supply a network of diverse, quality respondents to their worldwide client base and, to date, has completed more than 15,000 projects across 2500 universities worldwide. Qualtrics invited young people aged 13–24 years, living in the UK and able to read and write in English to take part in an online survey. No other exclusion criteria were applied. Qualtrics and their partners typically use a variety of channels to contact prospective participants, including, emails, SMS and in-app notifications. All participants over 18 years old and parents/guardians of participants under 18 years old provided informed consent to participate in the study. Participants under 18 years old provided informed assent after parental consent had been given. As part of the consent and assent processes, participants and parents were informed about the purpose of the study, that their/their child’s data would be treated in confidence and of their/their child’s right to terminate the study at any time without giving a reason.

As a result of the processes used by Qualtrics and partners to recruit respondents, it was not possible to determine the number of survey invitations that were distributed to panel members, or the proportion of panellists who were alerted to the survey and who did or did not complete the survey (i.e., the response rate). In total, 2050 participants completed the survey in full, however, 48 responses were removed for being over aged 24 years. Due to few respondents endorsing any gender categorisation other than male or female, and the use of gender as a predictor in the regression model, these responses (nonbinary, other and prefer not to say) were treated as missing data for the purposes of the current study. This resulted in a final sample of 1971 young people aged 13–24 who completed the survey.

### Measures

#### Anxiety and depression

The Hospital Anxiety and Depression Scale (HADS) [30] is a 14-item self-rating scale developed to measure anxiety and depression symptomology. It was specifically developed to avoid reliance on aspects of these conditions that are also common somatic symptoms of illness, for example, fatigue and insomnia or hypersomnia [30]. Participants were asked how often, over the past week, they had experienced seven anxiety symptoms and seven depressive symptoms. Each item was scored on a 4-point scale, ranging from 0 (absence of symptoms) to 3 (frequent symptoms). Each HADS subscale score ranges from 0 to 21, with higher scores indicating more considerable levels of anxiety or depression symptomology. The HADS has been found to have good reliability and discriminant validity [31]. The HADS has been validated for use with adolescents [32, 33]. Depression and anxiety caseness were classified using the cut-off scores suggested by White et al. [33]. For the anxiety subscale, scores 9–11 were classified as a ‘possible’ case, and scores ≥ 12 as a ‘probable’ anxiety case. For the depression subscale, scores 7–9 were classified as ‘possible’ depression, while ≥ 10 were considered as a ‘probable’ depression case. The internal reliability of anxiety (α = 0.82) and depression (α = 0.71) subscales in this sample was good.

#### COVID-19 related PTSD

Posttraumatic stress symptomology as a result of the COVID-19 pandemic was assessed using an adapted version of the Child Revised Impact of Events Scale (CRIES-8) [34, 35]. The CRIES-8 was specifically designed as a brief child-friendly screener for children at risk of PTSD, using eight items taken from the original Impact of Events Scale [36], designed to measure avoidance and intrusion symptomology. Participants were asked to rate the frequency of occurrence of these symptoms over the past week, with specific reference to the COVID-19 pandemic. Previous reference to “it” (i.e., the trauma) was replaced with “coronavirus”, which was the common terminology used at the time. For example, “Do you think about coronavirus even when you don’t mean to?” Response options were ‘None’, ‘Rarely’, ‘Sometimes’, and ‘Often’, scored as 0, 1, 3 and 5, respectively. Scores range from 0 to 40 with a cut-off score of 17 or above being used to classify PTSD caseness [35]. The CRIES has been found to have good psychometric properties when used as a screening tool for children exposed to a wide range of traumatic events [35, 37, 38]. The internal reliability of CRIES-8 scores in this sample was good (α = 0.79) and similar to that of previous studies using the original version of the measure [37, 38].

#### Wellbeing

The Short Warwick-Edinburgh Mental Well-being Scale (SWEMWBS) [39] is a 7-item unidimensional scale which has robust measurement properties for monitoring mental well-being and functioning in population surveys. Respondents are asked to rate the frequency of occurrence of seven positively worded statements (e.g., ‘I’ve been dealing with problems well’) with reference to the last two-weeks. All items are scored on a 5-point Likert scale, ranging from 1 ‘none of the time’ to 5 ‘all of the time’. Raw scores are converted into metric scores [40] which range from 7 to 35, with higher scores indicating overall better mental wellbeing. The SWEMWBS has been validated for use with adolescents [41, 42] The internal reliability of the SWEMWBS in this sample was good (α = 0.82).

#### Resilience

Child and Youth Resilience Measure (CYRM-R) is a self-report measures of social-ecological resilience designed for use with children and young people aged 5–23 years [43, 44]. The scale consists of 17 positively worded items, scored on a 5-point Likert scale ranging from 1 ‘Not at all’ to 5 ‘A lot’. The 17 items can be summed to produce a total score (range: 17–85). In addition, scores can be derived for two subscales of the CYRM-R: (1) personal resilience, which includes ten items related to intrapersonal and interpersonal experiences (range: 10–50), for example “I get along with people around me”, “I feel supported by my friends” and (2) caregiver resilience, which includes seven items in which the young person is asked to reflect on important caregiver relationships (range: 7–35), for example “I talk to my family/caregiver(s) about how I feel”, “I feel safe when I am with my family/caregiver(s)”. For both the overall total and the subscales, higher scores reflect higher levels of resilience. The psychometric properties of the scale have been well established [43]. The internal consistency of the overall total score (α = 0.92) and both the personal (α = 0.87) and caregiver resilience (α = 0.86) subscales were good in this sample.

#### Predictor variables

A number of categorical sociodemographic and continuous psychosocial variables were used to predict patterns of mental health in young people at the beginning of the COVID-19 pandemic:

#### Sociodemographic variables:

Demographic variables of *age* (categories: 13–15, 16–18, 19–21 and 22–24 years old)*, gender* (0 = female; 1 = male).

*Ethnicity* Ethnicity was recoded into a binary variable (0 = White, 1 = Black/African/Caribbean/Black British, Asian/Asian British, Mixed/Multiple ethnic groups or Other ethnic group). Responses of ‘prefer not to say’ were recoded and treated as missing data.

*Urbanicity* Participants were asked “Do you consider yourself to live in:” and were required to choose one of the options provided: ‘City’, ‘Suburb (a residential outlying district of a city)’, ‘Town’, or ‘Rural (countryside)’. The variable was recoded to a binary variable representing urbanicity (0 = City; 1 = Suburb, Town, or Rural).

*Home garden* Participants were asked if the place they lived had a garden (1 = Yes, 0 = No).

*Home privacy* Participants were asked about the amount of privacy they have in their home, using the following item: ‘We would like to know if where you live there is any space for you to be on your own, for some quiet time or for some privacy. For example, when you want to talk/hang out with your friends via social media/play on X-box etc.’ and were required to choose one of the response options provided ‘Yes’, ‘Sometimes’ or ‘No’. This was recoded into a binary variable (1 = Yes/Sometimes, 0 = No).

*Parental keyworker status* Participants were provided with a definition of keyworkers as people whose jobs are vital to public health and safety during the coronavirus lockdown and with examples of these types of jobs. They were then asked whether one, both or neither of their parents/caregivers were keyworkers. This was recoded in a binary variable indicating whether one or more parents/caregivers were key workers (1 = Yes, 0 = No).

#### Psychosocial variables

*Somatic symptoms* The Somatic Symptom Scale (SSS-8) [45] is a brief self-report questionnaire which was used to assess for the presence of somatic symptomology among the sample. It measures the perceived burden of common somatic symptoms (e.g., headaches, bowel/stomach problems, etc.). Respondents rate how much they were bothered by common somatic symptoms within the last seven days on a 5-point Likert scale, ranging from 0 ‘Not at all’ to 4 ‘Very much’. SSS-8 scores range from 0 to 32, with scores of ≥ 4, ≥ 8, ≥ 12, and ≥ 16 representing low, medium, high and very high levels of somatic symptomology severity, respectively [45]. The scale has been validated for use with individuals aged 13 and older [45]. Good internal consistency was found for the SSS-8 in the current sample (α = 0.87).

*Family Functioning* The McMaster Family Assessment Device is a self-report questionnaire used by clinicians and researchers to evaluate family functioning [46]. It is based on the McMaster Model of Family and includes a 12-item General Functioning (GF) subscale, which measures overall family functioning. The GF items describe both healthy and unhealthy aspects of family functioning related to problem solving, communication, roles, affective responsiveness, affective involvement, and behavioural control [47]. Items are scored on a 4-point Likert scale ranging from 1 ‘Strongly agree’ to 4 ‘Strongly disagree’, with negatively worded items reverse scored. A total score is produced by summing the 12 items, with higher scores reflecting poorer family functioning. The GF subscale has been validated as a single subscale measuring overall family functioning and good psychometric properties have been reported [48]. The GF subscale has previously been used to assess family functioning in children [49]. Internal consistency for the GF subscale was good in this sample (α = 0.85).

*Social networks* The short 6-item version of the Lubben Social Network Scale (LSNS-6) [50] was used to measure social contact and engagement with family and friends. Participants are asked how many (i) relatives and (ii) friends they see or hear from at least once a month, feel close to such that they could call on them for help, and with whom they feel at ease to take about private matters. Responses are scored on a 6-point Likert scale ranging from 0 ‘None’ to 5 ‘Nine or more’. All six items can be summed to produce a total score ranging from 0 to 30, with higher scores indicating greater levels of social contact. In addition, separate subscale totals can be calculated for family engagement (range: 0–15) and friend engagement (range: 0–15). Good psychometric properties have been reported for the overall scale as well as the two subscales [51]. Good internal consistency was found for the overall scale (α = 0.80), and the family (α = 0.77) and friends (α = 0.83) subscales in the current study.

*Attachment* The Relationships Questionnaire (RQ) [52], is a 5-item measure designed to describe prototypical attachment patterns in close adult relationships. Firstly, participants are presented with four short paragraphs briefly describing secure, fearful, preoccupied and dismissing attachment styles and asked to rate on a 7-point Likert scale from 1 ‘Strongly disagree’ to 7 ‘Strongly agree’ how well that attachment style describes their close relationships, providing a profile of their attachment style. An additional fifth item, then asks the participants to choose which of the four attachment styles best describes them. The ‘Model of Self’ subscale is an attachment pattern characterised by positive self-models (secure + dismissing) minus patterns characterised by negative self-models (fearful + preoccupied). The ‘Model of Other’ subscale is an attachment pattern characterised by positive other models (secure + preoccupied) minus patterns characterised by negative models of others (fearful + dismissing). These subscales are calculated using the continuous attachment pattern scores, in which higher scores reflect more positive models of self/others and range from − 12 to 12 [53].

*COVID-19 anxiety* The survey included a question “How anxious are you about the coronavirus COVID-19 pandemic?” and the participants were provided with a ‘slider’ (electronic visual analogue scale) to indicate their degree of anxiety with 0 indicating ‘not anxious at all’ and 100 indicating ‘extremely anxious’ at the left- and right-hand extremes respectively. This produced continuous scores ranging from 0 to 100 with higher scores reflecting higher levels of COVID-19 related anxiety.

### Analytic plan

While much of the previous dual-factor research has employed theoretically-driven approaches (e.g. cut-off scores/caseness) to classify participants, in recent years there has been a move towards categorising participants using exploratory empirical methods, such as LPA. LPA is a statistical method used to identify homogeneous groups or classes, from continuous data. It is a person-centred approach which classifies participants based on the data rather than confirming a predefined theoretical model. Although there are strengths and limitations to both theoretical and empirical methods (see [24, 54]), the current study used LPA to attempt to capture the nuances of mental health during a period of great uncertainty for UK adolescents.

Firstly, LPA was conducted to identify subgroups of young people with similar mental health profiles using scores for anxiety, depression, COVID-19 trauma, wellbeing, personal resilience and caregiver resilience. The fit of six models (a 1-class through a 6-class model) was assessed. The models were estimated using robust maximum likelihood [55]. To avoid solutions based on local maxima, 100 random sets of starting values were initially used, with 10 final stage optimisations. The relative fit of the LPA models were compared by using three information theory-based fit statistics: the Akaike information criterion (AIC) [56], the Bayesian information criterion (BIC) [57] and the sample size-adjusted Bayesian information criterion (ssa-BIC) [58]. The model that produced the lowest values was judged to be the best fitting model. However, the BIC is considered to be the best of the fit indices for deciding the number of classes in LPA [59]. In addition, the Lo-Mendell-Rubin likelihood ratio test (LRT) [60] was used to compare models with increasing numbers of latent classes. When the LRT becomes non-significant, it suggests the model with one less class is a better fit to the data. LPA models were estimated using Mplus 7 [61]. After determining the optimal number of classes, Vermunt’s three-step approach [62] was used to conduct a multinomial logistic regression (odds ratios and 95% confidence intervals) and identify which sociodemographic and psychosocial variables significantly predicted class membership.

## Results

### Sample characteristics

The majority of the sample were female (*n* = 1301, 66%) and of White ethnicity (*n* = 1498, 76.6%). The mean age of the sample was 18.64 years (*SD* = 3.17; range: 13–24 years). Around three-quarters of the sample lived with their parent(s)/caregiver(s)/family (*n* = 1482, 75.2%) and approximately two-thirds of the sample were attending school, college or university (*n* = 1305, 66.2%). Living in a town was the most common place of residence (*n* = 633, 32.1%), compared to living in a city (*n* = 572, 29.0%), suburb (*n* = 525, 26.6%) or rural area (*n* = 241, 12.2%). Descriptive statistics for sociodemographic and psychosocial characteristics of the sample can be found in Table 1.

**Table 1:** Sociodemographic and psychosocial characteristics of the sample (*N* = 1971)

	*N*	%
Age		
13–15	386	19.6
16–18	615	31.2
19–21	515	26.1
22–24	455	23.1
Gender		
Female	1301	66.0
Male	670	34.0
Ethnicity		
White	1498	76.0
Other ethnicity	457	23.2
Urbanicity		
City	572	29.0
Suburb/town/rural	1399	71.0
Home garden		
No	250	12.7
Yes	1721	87.3
Home privacy		
No	118	6.0
Yes/Sometimes	1842	93.5
Parent/caregiver(s) key workers		
No	891	45.2
Yes	1080	54.8
	*M*	*SD*
Psychosocial variables		
HADS: Anxiety (range: 0–21)	9.43	4.32
HADS: Depression (range: 0–21)	7.28	3.71
CRIES-8: COVID-19 traumatic stress (range: 0–40)	17.11	8.84
SWEMWBS: Wellbeing (range: 7–35)	19.57	3.72
CYRM-R: Personal resilience (range: 10–50)	36.65	7.85
CYRM-R: Caregiver resilience (range: 7–35)	26.33	6.32
LSNS-6: Family social engagement (range: 0–15)	7.20	3.10
LSNS-6: Friend social engagement (range: 0–15)	7.23	3.49
McMaster scale: Family functioning (range: 12–48)	25.70	6.06
SSS-8: Somatic symptoms (range: 0–32)	8.26	7.03
RQ: Attachment–Model of self (range: − 12–12)	0.45	3.76
RQ: Attachment–Model of Other (range: − 12–12)	− 0.47	3.39
COVID-19 anxiety (range: 0–100)	62.13	25.70

Percentages may not total 100% on demographic variables due to missing data

Based on the cut-off scores for the HADS, over a quarter of the young people in the study (27.1%) had ‘possible’ anxiety, while an additional third (32.0%) had ‘probable’ anxiety. Similar results were obtained for depression caseness with just under a third being categorised as ‘possible’ (29.1%) and ‘probable’ (29.2%) cases. Over half of the sample (53.9%) met the caseness for COVID-19 related PTSD based on the CRIES-8 scale cut-off. Mean wellbeing score was 19.57 (*SD* = 3.72). The prevalence of probable anxiety, probable depression and COVID-19 PTSD caseness broadly correspond to what has been reported elsewhere [63]. Correlations between the LPA indicator variables are included in Online Resource 1. Notably, COVID-19 traumatic stress had a small, but positive, association with both personal and caregiver resilience.

### LPA

**Fig. 1:**
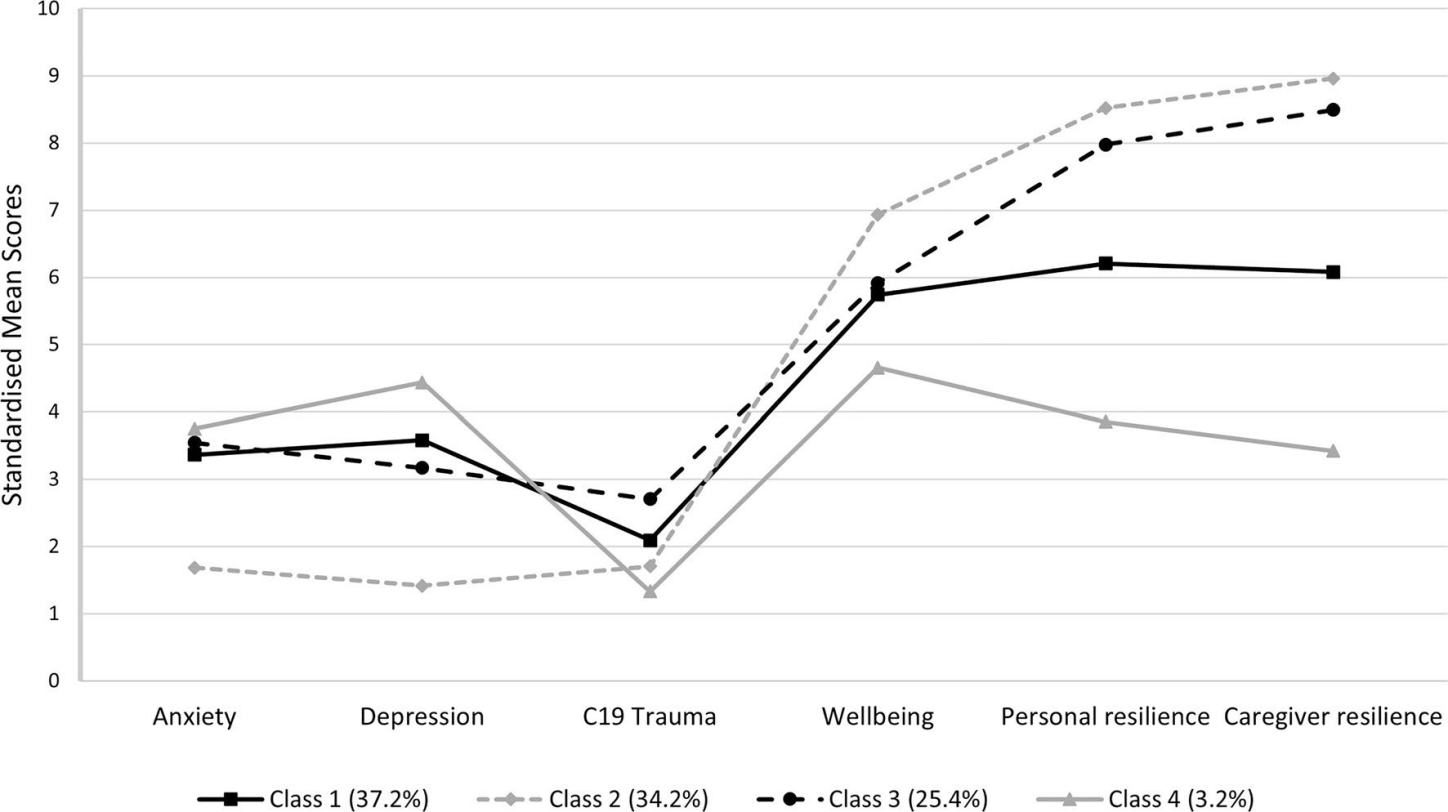
4-class latent profile analysis plot modelled using psychopathology and wellbeing indicators (standardised scores)

Fit indices for the LPAs are shown in Table 2. The AIC, BIC and ssaBIC continued to decrease from the 1-Class model through to the 6-Class model. The LRT, however, became non-significant in the 5-Class model, suggesting that the 4-Class model should be accepted. This solution also indicated acceptable classification of participants (entropy = 0.81). This solution revealed four distinct patterns of distress and wellbeing within the sample of young people (see Fig. 1).

**Table 2:** Fit statistics of latent profile analysis of young people’s (aged 13–24) mental health and wellbeing indicators (*N* = 1971)

Class	Loglikelihood	AIC	BIC	ssa-BIC	Entropy	LRT, *p*
1	− 36818.272	73660.545	73727.581	73689.456		
2	− 35545.939	71129.877	71236.017	71175.653	0.809	2497.64, < 0.001
3	− 35154.795	70361.590	70506.833	70424.230	0.762	767.82, < 0.001
**4**	**− 34930.528**	**69927.056**	**70111.404**	**70006.561**	**0.812**	**440.244, 0.035**
5	− 34801.229	69682.458	69905.910	69778.828	0.806	253.819, 0.487
6	− 34700.153	69494.306	69756.862	69607.541	0.775	198.42, 0.122

Selected model in bold

*AIC* Akaike information criterion, *BIC* Bayesian information criterion, *ssaBIC* sample size-adjusted BIC, *LRT* Lo–Mendell–Rubin-adjusted likelihood ratio test

**Fig. 2:**
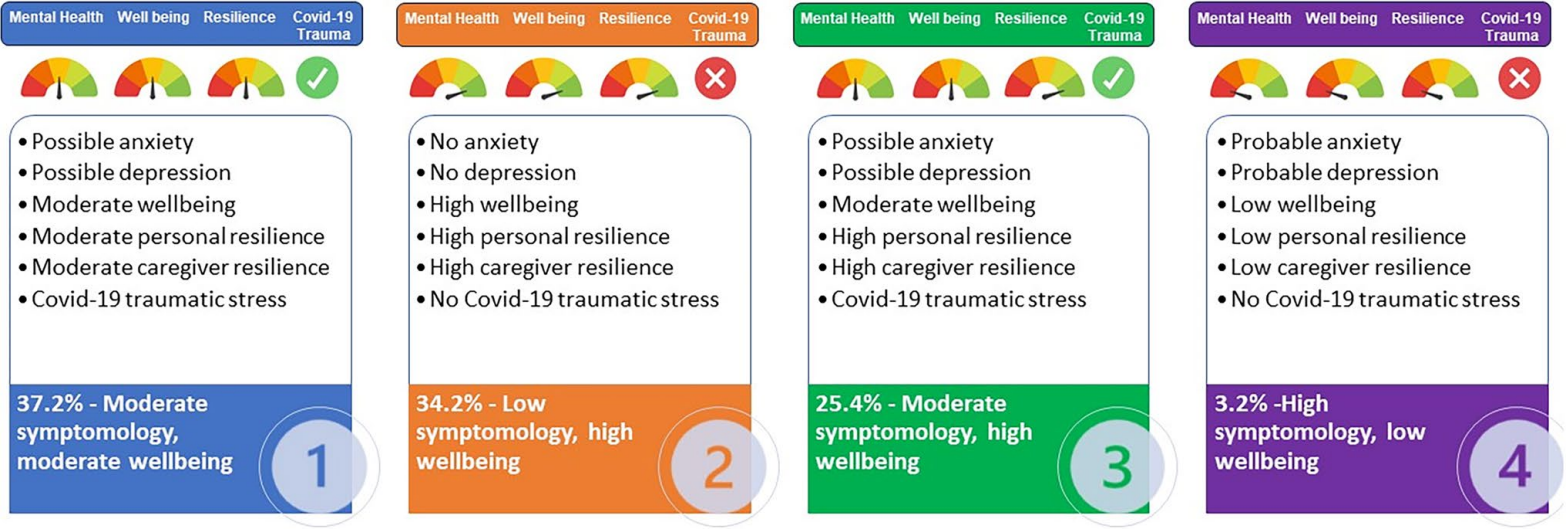
Summary of class characteristics

Class 1 (*moderate symptomology, moderate wellbeing*; *n* = 733, 37.2%) was the largest class and comprised a group of young people with moderate-to-high levels of symptomology, but also moderate levels of wellbeing. Their mean scores indicated they met caseness for ‘possible’ anxiety and depression, as well as meeting the threshold for COVID-19 related PTSD. Class 2 (*n* = 674, 34.2%) consisted of a group of young people with *low symptomology and high wellbeing*. They had the highest levels of wellbeing and both personal and caregiver resilience, and their mean scores suggested that they did not meet the caseness criteria for anxiety, depression or PTSD. Based on the mean scores, Class 3 (*moderate symptomology, high wellbeing*, *n* = 500, 25.4%) had ‘possible’ anxiety and depression. They also met PTSD caseness and had the highest trauma score of all the groups. Additionally, similar to Class 2, they had high levels of wellbeing and resilience. Class 4 (*high symptomology, low wellbeing*, *n* = 64, 3.2%) was the smallest, and comprised a group of young people whose profiles indicated they had ‘probable’ anxiety and depression and low personal and caregiver resilience. However, this class also had moderate levels of wellbeing and the lowest scores for COVID-19 trauma. Class characteristics are summarised in Fig. 2 and additional class-specific descriptive statistics can be found in Online Resource 1.

### Predicting class membership

Odds ratios (ORs) for the sociodemographic and psychosocial variables predicting class membership are shown in Table 3. Class 2, *low symptomology and high wellbeing,* was used as the reference class. Experiencing *moderate symptomology and moderate wellbeing* (Class 1) was associated with being male (OR = 1.72), lower levels of social engagement with family (OR = 0.87) and friends (OR = 0.83), poorer family functioning (OR = 1.54), higher levels of somatic symptomology (OR = 1.19), a less positive model of self (OR = 0.85), and higher levels of COVID-19 anxiety (OR = 1.01). Older age, specifically being aged 19–21 years (OR = 2.24) and aged 22–24 years (OR = 3.98) compared to age 13–15 years, was also associated with Class 1 membership. Compared to Class 2, the *moderate symptomology and high wellbeing* class (Class 3) was associated with being aged 22–24 years (OR = 3.18), lower levels of social engagement with friends (OR = 0.91), poorer family functioning (OR = 1.18), higher levels of somatic symptoms (OR = 1.19), a less positive model of self (OR = 0.84) and higher levels of COVID-19 anxiety (OR = 1.04). Finally, the *high symptomology, low wellbeing* class (Class 4) was associated with being male (OR = 4.98), lower levels of social engagement with family (OR = 0.68) and friends (OR = 0.62), poorer family functioning (OR = 1.81), higher levels of somatic symptoms (OR = 1.23), and a less positive model of self (OR = 0.74).

**Table 3:** Multinomial logistic regression predicting class membership (odds ratios; ORs) (*N* = 1945)

	Class 1 (37.2%) Moderate symptomology, moderate wellbeing	Class 3 (25.4%) Moderate symptomology, high wellbeing	Class 4 (3.2%) High symptomology, low wellbeing
OR (95% CI)			
Age			
13–15	–	–	–
16–18	1.39 (0.75–2.56)	0.98 (0.54–1.76)	1.67 (0.36–7.68)
19–21	2.24 (1.19–4.25)*	1.51 (0.82–2.79)	2.05 (0.39–10.95)
22–24	3.98 (1.97–8.05)***	3.18 (1.69–5.97)*	2.21 (0.45–10.92)
Gender			
Female	–	–	–
Male	1.72 (1.12 – 2.64)*	0.76 (0.47 – 1.23)	4.98 (1.78 – 13.98)***
Ethnicity			
White	–	–	–
Other ethnicity	0.94 (0.57–1.55)	0.61 (0.35–1.07)	0.38 (0.14–1.06)
Urbanicity			
City	–	–	–
Suburb/town/rural	0.92 (0.57–1.49)	0.87 (0.53–1.42)	0.49 (0.19–1.27)
Home garden			
No			
Yes	0.88 (0.47–1.67)	1.57 (0.78–3.16)	0.72 (0.22–2.38)
Home privacy			
No			
Yes/Sometimes	1.30 (0.44–3.90)	1.62 (0.48–5.45)	1.01 (0.22–5.60)
Parent/Caregiver(s) key workers			
No			
Yes	1.03 (0.68–1.54)	1.00 (0.66–1.53)	0.71 (0.30–1.69)
Psychosocial variables			
Family social engagement	0.87 (0.81–0.93)***	1.00 (0.93–1.07)	0.68 (0.56–0.82)***
Friend social engagement	0.83 (0.78–0.88)***	0.91 (0.85–0.97)***	0.62 (0.51–0.75)***
Family functioning	1.54 (1.45–1.65)***	1.18 (1.12–1.25)***	1.81 (1.60–2.03)***
Somatic symptoms	1.19 (1.14–1.24)***	1.19 (1.14–1.24)***	1.23 (1.15–1.32)***
Attachment–Model of self	0.85 (0.80–0.90)***	0.84 (0.78–0.89)***	0.74 (0.65–0.85)***
Attachment–Model of Other	0.97 (0.92–1.03)	0.97 (0.91–1.03)	1.00 (0.86–1.17)
COVID-19 anxiety	1.01 (1.01–1.02)***	1.04 (1.03–1.05)***	1.00 (0.98–1.02)

Class 2 (34.2%; *low symptomology, high wellbeing*) is reference class. Significant ORs in bold, *p < 0.05, **p < 0.01, ***p < 0.001

## Discussion

The current study aimed to provide a comprehensive examination of adolescent mental health during the initial peak phase of the COVID-19 pandemic in the UK. To do so, an exploratory LPA was conducted, using indicators of psychological distress and psychological wellbeing to identify homogeneous mental health groups in the population and examine the predictors of class membership. Of the classes that emerged from the best-fitting model (i.e., a 4-class solution), three of these mapped onto the groups outlined in the dual continua model, reflecting *high symptomology, low wellbeing* (Class 4, 3.2%), *low symptomology, high wellbeing* (Class 2, 34.2%); and *moderate symptomology, high wellbeing* (Class 3, 25.4%) groups. A ‘vulnerable’ (i.e., low symptomology, low wellbeing) group was not identified and instead, a class characterised by *moderate symptomology and moderate wellbeing* emerged as the largest class (Class 1, 37.2%).

Due to differences in samples and indicators used in previous studies, it is difficult to make direct comparisons; however, the current study closely aligns with that of Moore et al. [24]. These authors found that a ‘moderate mentally healthy’ class was the most populated amongst a sample of US high school students, and additionally, that both the ‘complete mental health’ (low symptomology, high wellbeing) and ‘symptomatic but content’ (high symptomology, high wellbeing) classes were also sizeable, each comprising around a third of the sample. Furthermore, comparability with previous research is limited given the unique impact of the COVID-19 pandemic and the disruptions that this caused for young people, particularly in relation to homeschooling and remote learning. Janousch and colleagues [28] examined the mental health of Swiss adolescents (aged 12–16 years) during the COVID-19 pandemic in August/September 2020 and similarly did not report the presence of a ‘vulnerable’ mental health class. This study used indicators of anxiety and depression (distress indicators), and personal competence, social competence, structured style, social resources and family cohesion (resilience indicators) and reported three distinct groups: resilient (high mental health issues, high protective factors, 26.3%), non-resilient (high mental health issues, low protective factors, 37.3%)**,** and untroubled (low mental health issues, high protective factors, 36.4%). These categories somewhat correspond to Class 3, Class 4, and Class 2 identified in the current study, respectively. Notably, however, the size of the equivalent *high symptomology, low wellbeing* class (Class 4) was much larger in the Swiss study. This may be a result of cultural or age differences, the lack of inclusion of specific psychological wellbeing indicators, or data collection at a later point during the pandemic compared to the current study.

We identified a small proportion of young people who were significantly distressed. The *high symptomology, low wellbeing* class was characterised by the highest scores for anxiety and depression and the lowest scores for wellbeing, personal resilience and caregiver resilience. Interestingly, however, it was also characterised by the lowest scores on COVID-19 trauma. Furthermore, unlike the *moderate symptomatology, moderate wellbeing* and *moderate symptomology, high wellbeing* classes, membership of this class was not significantly associated with COVID-19 anxiety. While the *high symptomology, low wellbeing* class having the lowest scores on COVID-19 trauma might initially seem counterintuitive, it may perhaps reflect substantive pre-existing mental health difficulties in this group of young people, overshadowing any additional (or specific) impacts brought on by the pandemic. Again, although speculative, this effect may reflect emotional numbing or dissociation observed in individuals suffering depression [64] and anxiety disorders, such as PTSD [65]. Emotional numbing is a process of shutting out feelings and may be experienced as a deficit of emotional response or reactivity, acting to block positive and negative emotions and experiences. The low COVID-19 specific associations in the *high symptomology, low wellbeing* class (i.e., COVID-19 trauma and COVID-19 anxiety), therefore, could be a consequence of emotional numbing in response to a global threat.

Compared to the *low symptomology, high wellbeing* class, all other classes were associated with lower social engagement with friends, poorer family functioning, greater somatic symptoms and a less positive model of self. Additionally, Classes 1 (*moderate symptomology, moderate wellbeing*) and 4 (*high symptomology, low wellbeing*) were associated with lower family social engagement. Adolescence is a period of increased need for social interaction, particularly with peers [6] and therefore, the increase in social deprivation as a result of social distancing and lockdowns during the pandemic may have particularly difficult. These findings align with the general mental health literature, which indicate a small, but positive, association between social support and children and adolescent wellbeing [66]. Moreover, previous dual-factor studies have reported low levels of peer social support are associated with poorer mental health during childhood, and additionally, with deteriorating mental health moving into adolescence [25].

The findings indicate that adolescents classified within the *low symptomology, high wellbeing* class (Class 2) demonstrated higher levels of social engagement during the lockdown period raises intriguing questions about the factors contributing to their resilience and adaptive coping strategies. Possible explanations may include increased reliance on online social networks, stronger community ties in rural areas, or proactive efforts to maintain social connections despite physical distancing measures. Understanding the behaviours and support systems that contribute to enhanced social engagement among resilient adolescents can inform interventions aimed at bolstering mental health during crises.

Furthermore, given that most adolescents in the study were living with their families, it is not unexpected that poor family functioning would be associated with the classes characterised by some degree of psychological distress. Specifically, COVID-19-related stressors have been found to be indirectly associated with anxiety through exacerbating family conflicts and eroding family cohesion [67] and poorer family functioning was associated with increased risk of depressive symptoms among adolescents [68]. Interestingly, compared to the *low symptomology, high wellbeing* (Class 2) group, membership of the *moderate symptomology, moderate wellbeing* (Class 1) and *high symptomology, low wellbeing* (Class 4) classes in this study were associated with male gender. This contrasts with previous studies which have found these groups to be more associated with females [23]. However, others have noted that these associations appear to be sensitive to culture and context, which may account for the inconsistent findings [69].

Our findings suggest that interpersonal skills, such as social engagement and family functioning, play a significant role in differentiating between mental health profiles among adolescents during the COVID-19 pandemic. Contrary to expectations, in our study, environmental factors like urbanicity or access to outdoor spaces like gardens did not emerge as strong predictors of mental health status [70]. This finding may be attributed to the unique circumstances of global challenges like a pandemic, where other factors may assume greater importance in shaping mental health outcomes. During periods of widespread uncertainty and disruption, adolescents may prioritize social connections, family support, and coping strategies over access to outdoor environments.

Interestingly, this study also revealed a notable association between age and mental well-being, with younger adolescents exhibiting better mental health outcomes compared to their older counterparts. These findings are consistent with a study by Morales-Vives et al. [71] who found higher levels of frustration and depressive symptoms, and lower levels of life satisfaction during the pandemic. Together these findings suggest a potential vulnerability among older adolescents (aged 19–24 years) during times of crisis, possibly due to increased academic, career, and financial pressures, as well as heightened awareness of societal challenges. Future research should explore the specific stressors faced by older adolescents and explore targeted interventions to support their mental health needs during and after crises.

Given the importance of social interaction during adolescent/young adulthood, alongside how dramatically lockdowns and social distancing regulations impacted most young people’s social networks, interventions targeting social connectedness may be of particular benefit. Previous research has suggested that individuals who were classified as ‘active and happy’ both during and post-lockdown were less likely to report changes in their social relations during this period, compared to those who were classified as inactive, distressed or lonely [72]. In this post-COVID era, social bonding interventions may help young people to maintain existing social relationships, build new meaningful connections, enhance resilience and decrease loneliness [73]. The results of the current study suggest that young men (aged 19–24) may benefit the most from targeted intervention related to social connection, aligning recent research finding that young men are most vulnerable to loneliness [74].

The current study used a person-centred, data-driven, exploratory approach to classify participants into mental health groups, allowing for a more sensitive analysis of adolescent mental health. Furthermore, it has added to the body of literature examining the dual-continua model among young people using LPA, rather than pre-determined cut-scores and similar confirmatory approaches. Moreover, the current study utilised data collected during a unique time: approximately one month after the UK government implemented the first lockdown and during the first peak of COVID-19 cases in the UK, giving a unique insight into how adolescents coped during this period of great uncertainty and change. However, there are several limitations which should be considered. Firstly, a broad definition of adolescence was used, capturing both teenagers and young adults. Although age significantly differentiated between classes, it is possible that separate analysis of younger and older adolescents may have yielded a different number and/or configuration of mental health classes. Secondly, mental health was examined cross-sectionally. As previously noted, longitudinal cohort studies have reported that psychological distress peaked during this initial phase of the pandemic and declined in the months following (e.g., [12]). Therefore, the current study can only examine adolescent mental health during this distinct time period but cannot examine the stability of these classes as the pandemic rapidly unfolded. Thirdly, only internalising symptoms of anxiety, depression and COVID-19 related traumatic stress were measured; changes in externalising and conduct behaviours were not modelled. Finally, much of the dual-continua research is rooted in an educational psychology context and includes academic indicators in their analyses. As such, it is important to note the wide variety of indicators used across studies.

## Conclusions

In conclusion, using indicators of distress, wellbeing and resilience, four classes of adolescent mental health were identified during the initial phases of the COVID-19 pandemic in the UK. The emergence of three of these classes (Classes 2, 3 and 4) were broadly in line with the dual-continua theory, while the emergence of Class 1 (*moderate symptomology, moderate wellbeing*) class highlights the nuances in mental health which cannot be observed by relying on theory-based methods alone. Furthermore, although around two-thirds of adolescents were showing moderate-to-high symptomology, the vast majority of these individuals also reported concurrent moderate-to-high levels of wellbeing, reflecting resilience at a time of extreme challenge. This counters the narrative that for most adolescents, high symptomology during this phase of the pandemic was indicative of poor mental health/disorder. Rather, we suggest that for many young people, it reflected an appropriate and adaptive response to the threat and challenges resulting from the COVID-19 pandemic. Furthermore, these findings demonstrate how a more comprehensive picture of mental health can be gained through adopting a dual-continua conceptualisation of mental health that incorporates both distress and wellbeing. In this way, at-risk youth can be identified and interventions and resources targeted appropriately.

### Supplementary Information

Below is the link to the electronic supplementary material.

Supplementary material file 1. Supplementary tables.

Supplementary material file 2. Dataset for current study.

## Data Availability

All data generated or analysed during this study are included in this published article and its supplementary information files.
