# Supplementary material for: Psychological distress, wellbeing and resilience: modelling adolescent mental health profiles during the COVID-19 pandemic
Source: Discov Ment Health. 2024 May 23;4(1):16. doi: 10.1007/s44192-024-00071-8 (PMC11116324; doi:10.1007/s44192-024-00071-8)
Supplement: Supplementary file 1 — Supplementary material file 1. Supplementary tables. [file 44192_2024_71_MOESM1_ESM.docx]

**Online Resource 1**

Online supplementary material for ‘Psychological distress, wellbeing and resilience: Modelling adolescent mental health during the COVID-19 pandemic’ submitted to *Discover Mental Health.* Corresponding author: Dr Sarah Butter ([s.butter@ulster.ac.uk](mailto:s.butter@ulster.ac.uk))

**Supplementary Table 1**. Bivariate correlations between distress, wellbeing and resilience variables included in the LPA (*N*=1971).

|  |  | 1 | 2 | 3 | 4 | 5 | 6 |
| --- | --- | --- | --- | --- | --- | --- | --- |
| 1 | Anxiety | - |  |  |  |  |  |
| 2 | Depression | 0.58 | - |  |  |  |  |
| 3 | COVID-19 traumatic stress | 0.38 | 0.22 | - |  |  |  |
| 4 | Wellbeing | -0.45 | -0.49 | -0.12 | - |  |  |
| 5 | Personal resilience | -0.32 | -0.48 | 0.08 | 0.39 | - |  |
| 6 | Caregiver resilience | -0.34 | -0.49 | 0.11 | 0.37 | 0.74 | - |

*Note*. All *p* <0.01

**Supplementary Table 2**. Class-specific descriptive statistics (*N*=1971).

|  | **Class 1 (37.2%)**  **Moderate symptomology, moderate wellbeing** | **Class 2 (34.2%)**  **Low symptomology, high wellbeing** | **Class 3 (25.4%)**  **Moderate symptomology, high wellbeing** | **Class 4 (3.2%)**  **High symptomology, low wellbeing** |
| --- | --- | --- | --- | --- |
| **Gender** |  |  |  |  |
| Male | 261 (35.6%) | 261 (38.7%) | 117 (23.4%) | 31 (48.4%) |
| Female | 472 (64.4%) | 413 (61.3%) | 383 (76.6%) | 33 (51.6%) |
|  |  |  |  |  |
| **Age** |  |  |  |  |
| 13 – 15 | 88 (12.0%) | 216 (32.0%) | 74 (14.8%) | 8 (12.5%) |
| 16 – 18 | 215 (29.3%) | 235 (34.9%) | 141 (28.2%) | 24 (37.5%) |
| 19 – 21 | 228 (31.1%) | 131 (19.4%) | 137 (27.4%) | 19 (29.7%) |
| 22 – 24 | 202 (27.6%) | 92 (13.6%) | 148 (29.6%) | 13 (20.3%) |
|  |  |  |  |  |
| **Mean (SD)** |  |  |  |  |
| HADS: Anxiety | 11.09 (3.34) | 5.50 (2.87) | 11.92 (3.40) | 12.48 (4.07) |
| HADS: Depression | 9.44 (2.74) | 3.62 (2.21) | 8.46 (2.50) | 11.78 (3.49) |
| CRIES-8: C-19 traumatic stress | 16.92 (8.08) | 13.84 (8.21) | 22.60 (7.70) | 10.80 (9.98) |
| SWEMWBS: Wellbeing | 18.26 (3.04) | 22.08 (3.34) | 18.77 (2.82) | 14.87 (4.92) |
| CYRM-R: Personal resilience | 30.73 (4.72) | 42.38 (4.97) | 39.91 (4.95) | 18.64 (6.03) |
| CYRM-R: Caregiver resilience | 20.95 (3.64) | 31.15 (3.17) | 29.64 (3.08) | 11.36 (3.08) |
|  |  |  |  |  |
| **Cut-off score (N, %)** |  |  |  |  |
| HADS: Anxiety |  |  |  |  |
| Normal | 146 (19.9%) | 576 (85.5%) | 75 (15.0%) | 8 (12.5%) |
| Possible case | 256 (34.9%) | 89 (13.2%) | 172 (34.4%) | 18 (28.1%) |
| Probable case | 331 (45.2%) | 9 (1.3%) | 253 (50.6%) | 38 (59.4%) |
| HADS: Depression |  |  |  |  |
| Normal | 101 (13.8%) | 611 (90.7%) | 107 (21.4%) | 4 (6.3%) |
| Possible case | 257 (35.1%) | 61 (9.1%) | 239 (47.8%) | 16 (25.0%) |
| Probable case | 375 (51.2%) | 2 (0.3%) | 154 (30.8%) | 44 (68.8%) |
| CRIES-8: C-19 traumatic stress |  |  |  |  |
| No | 329 (44.9%) | 429 (63.6%) | 104 (20.8%) | 47 (73.4%) |
| Yes | 404 (55.1%) | 245 (36.4%) | 396 (79.2%) | 17 (26.6%) |
